# Medication nonadherence and associated factors in patients with tuberculosis in Wau, South Sudan: a cross- sectional study using the world health organization multidimensional adherence model

**DOI:** 10.1186/s13690-024-01339-9

**Published:** 2024-07-15

**Authors:** Peter Michael Marin, Musso Munyeme, Clovice Kankya, Ambrose Samuel Jubara, Enock Matovu, Peter Waiswa, Javier Sanchez Romano, Francis Mutebi, David Onafruo, Estella Kitale, Owori Benard, Kayla J. Buhler, Morten Tryland

**Affiliations:** 1 Department of Public Health, University of Bahr El Ghazal (UBG), Wau, South Sudan; 2 Department of Disease Control, University of Zambia, Lusaka, Zambia; 3 Department of Biosecurity, Ecosystem and Veterinary Public Health, Makerere University, Kampala, Uganda; 4 Department of Clinical Studies, University of Bahr El Ghazal (UBG), Wau, South Sudan; 5 Department of Bimolecular Resources and Biolab Sciences, Makerere University, Kampala, Uganda; 6 Department of Medical Biology, UiT-The Arctic University of Norway (UiT), Tromsø, Norway; 7 Department of Pharmacy, Clinical and Comparative Medicine, Makerere University, Kampala, Uganda; 8 Clinical Epidemiology Unit, Makerere University, Kampala, Uganda; 9 Department of Forestry & Wildlife Management, Inland Norway University of Applied Sciences (INN), Evenstad, Norway; 10 Department of Arctic & Marine Biology, UiT - The Arctic University of Norway (UiT), Tromsø, Norway

**Keywords:** Urine isoniazid testing, Mycobacterium tuberculosis, Counseling, Health education, Public Health

## Abstract

**Background:**

Tuberculosis medication nonadherence is a multi-dimensional public health problem with serious consequences worldwide. There is little information available for medication nonadherence in South Sudan. This study assessed the proportion, reasons, and associated factors for nonadherence among patients with TB in Wau Municipality, South Sudan.

**Methods:**

A health facility based cross-sectional study was conducted among 234 tuberculosis (TB) patients receiving first line anti-TB regimen in Wau Municipality. Urine isoniazid metabolite testing (IsoScreen^®^) was used to determine nonadherence (visualized by negative test results) and a questionnaire was used to describe the reasons for nonadherence. Modified poisson regression with robust standard errors was performed since the proportion of nonadherence was < 10%, to identify nonadherence associated factors using the WHO Multidimensional adherence model.

**Results:**

Out of 234 participants, 24.8% (95% CI, 19.2 − 30.3) were nonadherent to the TB treatment regimen. At multivariate analysis, nonadherence was significantly associated with: relief of symptoms (APR 1.93, 95% CI 1.12 − 3.34, *p* = 0.018), alcohol use (APR 2.12, 95% CI 1.33 − 3.96, *p* = 0.019) and waiting time to receive drugs (APR 1.77, 95% CI 1.11 − 2.83, *p* = 0.017).

**Conclusion:**

Tuberculosis medication nonadherence was high, and it’s associated with patients’ relived of symptoms, alcohol use, and prolonged waiting time at health facility. Hence, addressing these barriers and the use of multifaceted interventions e.g. counseling, health education and improve appointments are crucial to reduce nonadherence among patients with TB in South Sudan.

**Supplementary Information:**

The online version contains supplementary material available at 10.1186/s13690-024-01339-9.

**Table:** 

**Text box 1. Contributions to the literature**
There is a gap in understanding the adherence and the World Health Organization Multidimensional adherence factors in TB medication.
TB medication adherence become more challenging among patients in a fragile context like South Sudan.
Stakeholders to pay attention to a multidimensional adherence factors in TB management to accelerate End of TB strategy.

## Background

Tuberculosis (TB), an infectious disease caused by the bacterium *M. tuberculosis*, is a major public health problem worldwide, with approximately 10.6 million cases and 1.6 million related deaths in 2022 [1–3]. In South Sudan, the disease is a leading cause of death, with the incidence rate reported as 79 cases per 100,000 sputum smears positive TB, and 140 cases per 100, 000 for all forms of TB [4]. Additionally, tuberculosis treatment success rate was 80–85%, and this is below the WHO acceptable threshold of at least 90% [5]. Furthermore, TB treatment interruptions, retreatment, multi-drug resistant TB (MDR-TB) and suboptimal treatment outcomes have all been reported in the country [6].

In patients with TB, the disease is curable with the current recommended six months treatment regimen using four first-line drugs (isoniazid, rifampicin, ethambutol and pyrazinamide). Without treatment, there is high mortality in patients. According to South Sudan’s TB treatment guideline, the treatment has two consecutive phases. In the initial (or intensive) phase, isoniazid, rifampicin, pyrazinamide, and ethambutol are administered in fixed dose drug combinations (FDCs) daily to the patient for two months. This is followed by the continuation phase, with isoniazid and rifampicin administered in FDCs daily to the patient for the remaining four months [7].

Despite the disease being treatable, nonadherence and suboptimal treatment outcomes rates are alarming among patients worldwide including in South Sudan [8, 9] .Therefore, this study is paramount, given the fact that, poor adherence may lead to serious public health consequences, including prolonged morbidity, community transmission, psychological distress, multi drug resistance (MDR), socio-economic barriers and death [10].

In the quest to understand adherence to TB and other long term therapies, the Multidimensional Adherence Model (MAM) was proposed by WHO, which describes adherence as the interaction of multiple factors, not merely a patient issue as considered before [11]. These factors were classified into 5 groups, including (1) socio-economic factors, (2) patients related factors, (3) clinical condition-related factors, (4) therapy-related factors and (5) healthcare team and systems factors [11].

In recent years, several studies have been conducted with the MAM model for different settings and chronic diseases, including haemophilia and HIV/AIDS [12–14]. In South Sudan, MAM has yet to be applied to identify factors associated with medication nonadherence among TB patients. Hence, this study assessed the proportion of nonadherence, described the reported reasons, and identified associated factors using MAM.

## Methods

### Study population and design

**Fig. 1:**
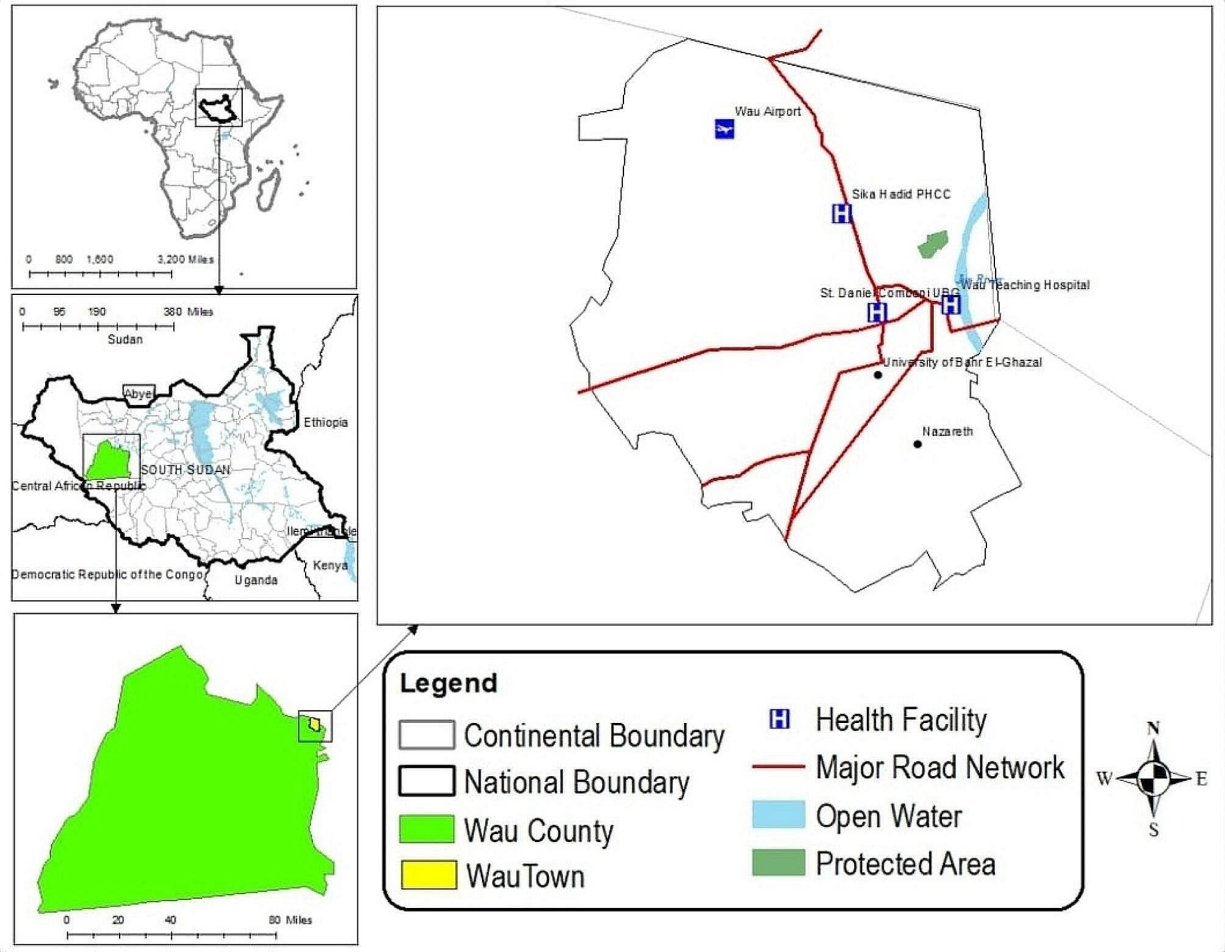
Study area map of Wau Municipality, South Sudan

A cross-sectional study was conducted in four health facilities in Wau Municipality, South Sudan, from 10th February to 20th June 2023. The town has a population of 127,384 people [15] and is located on the western banks of the Jur River (7°42’N, 27°59’E) (Fig. 1). The selected health facilities were chosen due to the availability of TB directly observed therapy services, including diagnosis and treatment. These services are integrated and provided free of charge by the Ministry of Health in partnership with health partners through directly observed treatment (DOT) sites, where patients come every month to receive a 28-days anti-TB medication quota to be administered at home under observation of their treatment supporter (health worker, family member or friend). Patients considered eligible for this study were pulmonary (any bacteriologically confirmed or clinically diagnosed case of TB involving the lung parenchyma or the tracheobronchial tree) and extra-pulmonary (any bacteriologically confirmed or clinically diagnosed case of TB involving organs or tissues other than the lung parenchyma) [7] adult patients under first line anti-TB treatment that had completed at least one month of treatment or were in the continuation phase (except for the final treatment month). Other inclusion criteria were that the patient had to be mentally sound, able to communicate and able to provide written informed consent for participation. Further exclusion criteria included those that were below 18 years of age, those with severe symptoms, those that did not pick their anti-TB drugs themselves, or those that declined to consent.

### Sample size and sampling design

Sample size (*n* = 234) was determined based on the below formula [16] using a 95% confidence interval, 5% margin of error, 10% expected non-response rate and considering 16.5% proportion of treatment nonadherence [17].

$${\mathbf{n}}{\text{ }} = {{\mathbf{Z}}^{\mathbf{2}}}{\mathbf{p}}{\text{ }}\left( {{\mathbf{1}} - {\text{ }}{\mathbf{p}}} \right)/{{\mathbf{d}}^{\mathbf{2}}}$$

**p =** proportion of treatment non-adherence (*p* = 16.5% (i.e. 0.165)) from previous similar study in Ethiopia.

**Z =** 1.96 at 95% confidence interval, **d =** expected margin of errors, i.e. 0.05,

**n** = required sample size, **10%** expected non-respondents rate.

The calculated sample size was (212 + 21 “10% non –respondent rate” = **233**) for patients.

However, the final sample size reached was 234 patients. All four health facilities, including three hospitals and one primary healthcare center that provided both tuberculosis diagnosis and treatment services, in the study area were selected. We proportionally identified from TB register books patients in intensive and continuation phases (2nd, 3rd, 4th, and 5th treatment months), and then continued enrollment of new patients initiating treatment until the required sample size was reached. The patients were randomly approached without prior notice during their monthly scheduled visit for interviews and urine samples at their contact health facility. The patients were interviewed about factors associated with nonadherence based on the MAM and reported reasons for nonadherence before urine samples were collected using round-bottom, screw-capped tube containers. Samples were stored in cool boxes and transported to the collection site in one of the health facilities for analysis within 3 h of collection. The study conducted a single urine sample collection and interviews without prior notice to minimize the likelihood of patients changing their adherence behavior in anticipation of the sampling.

### Urine isoniazid testing

Nonadherence was measured using the urine isoniazid metabolite test (IsoScreen^®^, GFC Diagnostics Ltd, England), an objective and validated test with high sensitivity and specificity [18]. The test is a point of care that takes 5–10 min to detect the presence of isoniazid or its metabolites in the patient’s urine and it does not require laboratory facility for analysis. As per the manufacturer instructions: The urine sample is measured by the syringe, which is then inserted into the tube and the cap pushed on to the barrel. Then, the action of pushing the cap on breaks the seals of two internal chambers which contain chemicals. These are pushed into the reaction chamber, to enable mixing all reagents together prior to activation. Finally, the sample is immediately injected into the reaction chamber. The sample is mixed with the reagents to assist dissolution. This is done by holding the cap firmly between thumb and finger and using the index (first) finger of the other hand to strike the bottom part of the barrel firmly to create a mixing or eddy motion within the fluid. If continued for about 20 s it will assist the reagents to dissolve.

Poor inter-rater agreement has been reported during interpretation in previous studies [19]. However, to minimize this impact, all the urine samples were assembled and analyzed in one selected health facility and the research team agreed on the classification of the color of the end result for each analyzed sample [19]. In this study, nonadherence was defined as negative, yellow test result (signifying a missing dose for the last 72 h or more) [20].

### WHO multidimensional adherence factors

Based on MAM (WHO) [11], a pretested and validated structured questionnaire was used to collect data on socio-economic, patient, therapy, condition and healthcare team and system factors.

Perceived stigma was measured using a validated 12 item stigma measurement scale [21]. The Kessler Psychological Distress 10 item Scale (K-10) tool was used to assess the level of psychological distress within the last month among the patients [22]. The WHO Alcohol Use Disorder Identification Test (AUDIT), 10 item questionnaire was used to describe alcohol use disorder [22]. Healthcare provider and patient relationship was measured using the Patient Reaction Assessment (PRA) 5 item scale [23] and the family and social support was assessed using a 6 item questionnaire previously used [24].

The reported reasons for nonadherence were collected from patients that were considered nonadherent using a Visual Analogue Scale (VAS) questionnaire, which is a validated tool for screening of patients in a resource limited setting [22], (the results were reported elsewhere).

### Data management and statistical analysis

All collected data were entered into EpiData version 3.1 then exported into STATA version 15 for analysis. Participant’s characteristics and reasons for nonadherence were presented using frequencies and percentages. A chi-square test was used to examine the relationship between categorical variables. Cronbach’s alphas tests were conducted to measure the internal reliability of the measurements, e.g. perceived stigma 11 item scale was (α = 0.85), the Kessler Psychological Distress 10 item Scale (K-10) was (α = 0.98), the WHO Alcohol Use Disorder Identification Test (AUDIT), 10 item questionnaire was (α = 0.93), Patient Reaction Assessment (PRA) 5 item questions was (α = 0.91), and the family and social support 6 item questions was (α = 0.94), where an alpha value ≥ 0.7% was considered satisfactory.

Modified poisson regression with robust standard errors was preferred to better predict the association when the prevalence of outcome is not rare (above 10%) and with a small sample size to avoid overestimation by the odds ratio (OR) [25]. Therefore, the Modified poisson regression with robust standard errors was used to assess the association between nonadherence and the suggested multidimensional adherence factors. Bivariate analysis were performed using family poisson and link log for which factors with *P* ≤ 0.2 were retained for multivariate model with a *P* ≤ 0.05 for retaining a variable in the last model. Interaction was assessed and potential confounders were adjusted. Adjusted prevalence ratios (APR) were calculated at 95% CI. In this questionnaire, four Likert’s scale questions were scored as follows: “strongly agree and agree” were scored with a one and “strongly disagree and disagree” were scored zero. Similarly, the five Likert’s scale were scored from 1 to 5 points. Consequently, perceived stigma cutoff above 6 was considered high perceived stigma [21], psychological distress cutoff above 25 was considered absent of psychological distress. Healthcare provider’s relationship cutoff above 7 was considered satisfactory, alcohol use risk cutoff above 8 was considered present of risk. In-addition, knowledge 10 items questions were calculated based on correct/incorrect answers, correct answers were scored one, incorrect answers were considered zero, and the cutoff above 5 was considered good knowledge. Economic status was assessed using 7 items ownership of basic assets. The responses to these questions were recorded by yes, that scored one and (no) that scored zero, the total scores were calculated and cutoff above 3 was considered as high economic status [22].

## Results

### Participant’s characteristics

Of the 282 eligible participants, 48 (17%) did not participate. This was due to family members collecting the drug for the patient, severe sickness, travel, or declining to participate. Participant characteristics are summarized in Tables 1 and 2. Nearly three quarters (*n* = 173, 73.9%) of the participants were males. Of the 234 patients, 103 (44%) had no formal education. Most patients had pulmonary tuberculosis (*n* = 227, 97%), 78 (33.3%) were in age category 18–28 years old, 142 (60.7%) were married, and 143 (61.1%) were pastoralists. Of the 234 patients, 197 (84.2%) and 175 (74.8%) had experienced perceived stigma and psychological distress, respectively. Only 11 (4.7%) of the participants had experienced harmful alcohol use.

The majority (*n* = 131, 56%) of participants were in the initiation (Intensive) treatment phase, spent less than 60 min waiting at healthcare facility to collect drugs (*n* = 189, 80.8%) and were satisfied with healthcare providers support (*n* = 233, 99.6%) (Tables 1 and 2).

### Proportion of nonadherence

Of the 234 patients that participated, 58 (24.8%) (95% CI, 19.2 − 30.3) were classified as nonadherent to medication.

**Table 1:** Socio-economic and patient related characteristics of the 234 TB patients included in the study

WHO adherence Dimension	Factors	Frequency	Percent (%)
**Socio-economic related factors**	**Education**		
	None	103	44.0
	Primary/Basic	81	34.6
	Secondary	42	17.9
	University	8	3.4
	**Economic status**		
	Low	234	100
	High	0	0.0
	**Occupation**		
	Formal employment	68	29.1
	Informal employment	166	70.9
	**Transport cost**		
	Yes	94	40.2
	No	140	59.8
	**Distance to HF**		
	0–5 km	204	87.2
	More than 5 km	30	12.8
	**Social Support**		
	Present	199	85.0
	Absent	35	15.0
**Patient related factors**	**Knowledge about adherence**		
	Poor	26	11.1
	Good	208	88.9
	**Perceived stigma**		
	Low	37	15.8
	High	197	84.2
	**Age**		
	18–28 years	78	33.3
	29–38 years	65	27.8
	39–48 years	45	19.2
	above 48 years	46	19.7
	**Sex**		
	Male	173	73.9
	Female	61	26.1
	**Marital Status**		
	Single	85	36.3
	Married	142	60.7
	Widowed	7	3.0
	**Ethnicity**		
	Dinka	143	61.1
	Fartit	64	27.4
	Lou/Jur	23	9.8
	Others	4	1.7

### Reported reasons for nonadherence among patients with TB

Of the 234 patients interviewed using the questionnaire, 29 (12.4%) reported missing more than 10% of doses in the last 30 days and were considered nonadherent. Among these, 7(24.1%) reported forgetfulness, and (20.7%, *n* = 6) reported traveling or being busy at work or school. Meanwhile, (17.2%, *n* = 5) of participants reported being hungry (no food available to swallow the drugs) and 3(10.3%) of patients reported nonadherent due to absence of the health worker or health facility closure during holidays (Table 3).

**Table 2:** Therapy, condition, health care team and system related characteristics of the 234 TB patients included in the study

WHO adherence Dimension	Factors	Frequency	Percent (%)
**Therapy related factors**	**Previous treatment failure**		
	Yes	21	9.0
	No	213	91.0
	**management of side effect**		
	Yes	208	88.9
	No	26	11.1
	**Treatment phase**		
	Intensive	131	56.0
	Continuation	103	44.0
	**Relief of symptoms**		
	Yes	26	11.1
	No	208	88.9
**Condition related factors**	**Severity of symptoms**		
	Yes	86	36.8
	No	148	63.2
	**Comorbidity (TB-HIV)**		
	Yes	10	4.3
	No	224	95.7
	**Alcohol use disorder**		
	Present	11	4.7
	Absent	223	95.3
	**Psychological distress**		
	Absent	59	25.2
	Present	175	74.8
	**Smoking history**		
	Yes	51	21.8
	No	183	78.2
	**Type of TB**		
	Pulmonary	227	97.0
	Extra-pulmonary	7	3.0
**Healthcare Team & system**	**Waiting time**		
	< than 60 min	189	80.8
	> than 60 min	45	19.2
	**Last counseling**		
	Last month	188	80.3
	More than month	46	19.7
	**HCP support satisfaction**		
	Satisfied	233	99.6
	Dissatisfied	1	0.4

**Table 3:** Patient reported reasons for nonadherence to TB medication in Wau, South Sudan (*n* = 29)

Reasons for nonadherence *	Proportion of patients reporting this reason** (*n*, %)
Forgetfulness	7(24.1%)
Travel	6(20.7%)
Busy (work/school)	6(20.7%)
Hunger (No food to swallow the drug)	5(17.2%)
Facility closed due to holidays	3(10.3%)
Absent of health worker	3 (10.3%)
No transport	1(3.4%)
Very sick	1(3.4%)

*nonadherence: patient reported missing more than 10% of doses using VAS.

**total patients reported nonadherence e.g. 29, some could report more than 1 reason.

### Factors associated with nonadherence to TB medication

In the bivariate analysis, several factors were considered to be associated with medication nonadherence across all five WHO multidimensional adherence dimensions at (*P* ≤ 0.2) (Table 4).

In the multivariate analysis, participants who were feeling relieved of TB symptoms were 1.93 times higher to be nonadherent (APR 1.93, 95% CI 1.12 − 3.34, *p* = 0.018), participants with alcohol use had 2.12 times higher nonadherence compared to those without (APR 2.12, 95% CI 1.13 − 3.96, *p* = 0.019), and participants who waited 60 or more minutes collecting drugs were 1.77 times higher to be nonadherent to medication (APR 1.77, 95% CI 1.11 − 2.83, *p* = 0.017) (Table 4).

**Table 4:** Modified Poisson regression analysis of WHO multi-dimensional factors associated with nonadherence to TB medication (*n* = 234)

WHO adherence Dimension	Factors	Nonadherence *n*, (%)	Bivariate results CPR(95% C I)	*P*-value	Multivariate results APR(95% C I)	*P*-value
**Socio-economic related factors**	**Distance to HF**					
	0–5 km	54(26.5)	1		1	
	More than 5 km	4(13.3)	1.99 (0.77 − 5.09)	0.154	2.19(0.88 − 5.46)	0.094
**Patient related factors**	**Knowledge**					
	Good	55(26.4)	1		1	
	Poor	3(11.5)	2.29(0.77 − 6.82)	0.136	2.43(0.84 − 7.01)	0.099
	**Smoking history**					
	No	41(22.4)	1		1	
	Yes	17(33.3)	1.49 (0.93 − 2.38)	0.100	1.32(0.81 − 2.14)	0.255
	**Alcohol use**					
	Present	6(54.5)	1		1	
	Absent	52(23.3)	2.34(1.29 − 4.22)	0.005*	2.11(1.33 − 3.96)	0.019*
**Therapy related factors**	**Treatment phase**					
	Continuation	30(29.1)	1		1	
	Intensive	28(21.4)	1.36(0.87 − 2.13)	0.174	0.75(0.48 − 1.16)	0.195
**Condition related factors**	**Relief of symptoms**					
	Yes	10(38.5)	1		1	
	No	48(23.1)	1.67 (0.96 − 2.88)	0.067	1.93(1.12 − 3.34)	0.018*
**Healthcare Team & system**	**Waiting time**					
	> than 60 min	15(33.3)	1		1	
	< than 60 min	43(22.8)	1.47(0.89 − 2.39)	0.127	1.77(1.11 − 2.83)	0.017*

*P* ≤ 0.2 were retained in bivariate and considered for multivariate analysis.

* P ≤ 0.05 were considered statistically significant at multivariate analysis.

## Discussion

The proportion of nonadherence found in our study, using a rigorous urine isoniazid metabolite testing was high. However, there are relatively few studies reported that have used this adherence measurement testing, making it more challenging to directly compare our findings on nonadherence to TB treatment with previous reports. The proportion of nonadherence found in our study was twice as high compared to the proportion reported in a study conducted in India [19], this may be due to the fact that the majority of the participants in the Indian study had HIV/TB co-morbidity and this condition required a consistent adherence to avoid serious complications.

Several factors across the MAM were significantly associated with nonadherence, which shed some light on the reasons for medication nonadherence in South Sudan [26]. Condition related factors, such as the relief of symptoms, was associated with nonadherence because patients stopped taking their medication when there was apparent improvement. In a study conducted among tuberculosis patients in Ethiopia, perceived wellness and cure were reported associated with nonadherence [27]. Similarly, Kardas et al. [28] found that the disappearance of symptoms lead to nonadherence among patients, which is in agreement with our findings. Thus, educating patients about the importance of adherence in spite of the relief of symptoms is paramount to ensure successful treatment outcomes [8, 19].

Furthermore, patients who used alcohol were two times higher to be nonadherent in this study. This is consistent with a study conducted in India [28], coupled with the fact that nearly three quarters (74.8%) of patients showed psychological distress. Studies have confirmed that alcohol use and psychological distress are factors associated with nonadherence to medication [29] and our findings highlight the importance of psychological support and counselling for TB patients during the prolonged treatment process [22]. Healthcare team and system related factors were also found to contribute to nonadherence. Long waiting times (approximately 60 min or more) at the health facility to collect monthly medication was significantly associated with nonadherence among patients, compared to waiting times that were less than 60 min. Patients who waited for 60 min or more had two times higher nonadherent. This is similar to studies conducted in India and Ethiopia [19, 27]. Therefore, healthcare providers are encourage to improve monthly appointments and reduce the waiting time at health facilities in accordance with a patient centered approach to TB care [10].

Finally, the major reported reasons for nonadherence among patients included forgetfulness, travelling time to the village, busy schedules (work or school), and hunger. These findings were consistent with studies conducted elsewhere. For example, in a qualitative study conducted in São Paulo health workers emphasized the role of food ration in improving adherence and successful treatment outcomes among patients [30]. Additionally, forgetfulness and travelling were reported among patients in Ethiopia and India [19, 31]. Likewise, lack of transportation means to collect monthly drugs was reported among reasons for nonadherence in Indonesia [9]. In our context, these reasons may be attributed to dire economic and social constraints faced by TB patients in South Sudan, which is a country that has recently emerged from prolonged wars and violence. Tailored economic and social support interventions may be needed to ensure appropriate adherence to treatment [19, 28]. Few studies have used the urine isoniazid testing, making it challenging to directly compare our findings with previous reports. The proportion of nonadherence found by this urine test in our study was twice as high as the proportion reported in a study conducted in India [19]. This may be due to the fact that the majority of Indian participants had HIV/TB co-morbidity, requiring consistent adherence to avoid serious complications contrary to our study. In addition, some participants reported intentionally avoiding some doses due to hunger and lack of food as mentioned earlier among the reasons for nonadherence in this study. In South Sudan, patients were previously receiving food assistance rations from humanitarian partners; however, this assistance is no longer available and may explain the high rate of nonadherence to treatment. This is supported by previous studies that have described food assistance to TB patients as enablers for the success of DOTs and adherence [32, 33].

Our study used an objective adherence measurement tool with high sensitivity and specificity. In addition, the use of MAM model gave us the broader perception of the complexity of nonadherence (instead of the single approach). However, when interpreting the results, it is important to keep in mind the limitations of a cross-sectional design. Generalizations are restricted to populations with similar parameters or risk factors. Besides, we found very few studies in the literature that had used urine isoniazid testing among TB patients, which made it difficult to directly compare the results in our study with previous reports.

Our findings may help with identifying patients at risk of nonadherence during routine care. Increased counselling, health education and prioritization of a patient-centered care approach by healthcare providers could improve adherence. Further studies are required on the interrelationships between MAM factors and nonadherence. In-addition, to understand the extent of zoonotic TB in patients, as pastoralists comprise about 60% of the current patients under treatment.

## Conclusions

The proportion of nonadherence is high, representing a challenge for public health with multiple associated factors. These include alcohol use, improve symptoms and waiting time at health facility. Addressing these barriers and the use of tailored multifaceted interventions such as: counseling, health education and improve appointments are crucial to reduce nonadherence among patients with TB in South Sudan.

### Electronic supplementary material

Below is the link to the electronic supplementary material.

Supplementary Material 1

Supplementary Material 2

Supplementary Material 3

Supplementary Material 4

Supplementary Material 5

## Data Availability

No datasets were generated or analysed during the current study.

## Abbreviations

TB: Tuberculosis
MDR-TB: Multi-drug resistant TB
FDC: Fixed-Dose Combination
WHO: World Health Organization
MAM: Multidimensional Adherence Model
APR: Adjusted Prevalence Ratio
DOT: Directly Observed Treatment
CPR: Crude Prevalence Ratio
