# Supplementary material for: Medication nonadherence and associated factors in patients with tuberculosis in Wau, South Sudan: a cross- sectional study using the world health organization multidimensional adherence model
Source: Arch Public Health. 2024 Jul 15;82:107. doi: 10.1186/s13690-024-01339-9 (PMC11250949; doi:10.1186/s13690-024-01339-9)
Supplement: Supplementary file 1 — Supplementary Material 1 [file 13690_2024_1339_MOESM1_ESM.pdf]

Date: 28/03/2024

To: Editor, BMC Public Health

Subject: Submission of the manuscript titled: **Medication nonadherence and associated factors in patients with tuberculosis in Wau, South Sudan: a cross- sectional study using the World Health Organization Multidimensional Adherence Model**

Dear Sir/Madam,

We would like to seize this opportunity to submit to your esteemed journal the above mentioned manuscript for publication. This research work is a contribution from South Sudan one of the underrepresented countries to the global science community. Furthermore the study is addressing tuberculosis, the disease of poverty and public health importance.

We hope that our research will shade the light on the problem of nonadherence to TB medication and associated factors in patients using one of the rigorous adherence measurement (urine isoniazid metabolite testing- IsoScreen), and the utilization of World Health Organization Multidimensional Adherence Model (MAM) in South Sudan settings.

This research work was conducted in the routine care setting contrary to the previous studies conducted at patient's homes. Our work is presenting new insight about possibility of using urine isoniazid metabolite testing in the routine care settings as recommended by the previously published study in India by **Ramnath Subbaraman and colleagues** in 2021.

Therefore, this is the original work submitted and we hope our contribution will present the prospective of the newest country to the global scientific literature. We are looking forward for your positive response, thanks.

Best regards,

Peter Michael Marin, corresponding author

PhD fellow at Makerere University, Uganda
