# Supplementary material for: Medication nonadherence and associated factors in patients with tuberculosis in Wau, South Sudan: a cross- sectional study using the world health organization multidimensional adherence model
Source: Arch Public Health. 2024 Jul 15;82:107. doi: 10.1186/s13690-024-01339-9 (PMC11250949; doi:10.1186/s13690-024-01339-9)
Supplement: Supplementary file 2 — Supplementary Material 2 [file 13690_2024_1339_MOESM2_ESM.pdf]

REPUBLIC OF SOUTH SUDAN

Ministry of Health, Research Ethics Review Board (MOH-RERB), Juba.

MOH/RERB/ Protocol: 8/31/01/2023 **Date:** 6th /02/2023

Project Title: "Elucidation of non-adherence to medication through A multi-dimensional practice; Case study of Tuberculosis treatment in South Sudan"

MOH/RERB/Approval/8/6/02/2023

Principal Investigator(s): Peter Michael Marin, MUK

MOH C**o-investigator(s)/collaborator(s):** Acier, MOH-Juba

Notice of Research Approval

This served to inform you that the research intervention described in the submitted protocol have been reviewed with comments, ethical opinions and suggestions to be harmonized by the Principal Investigator and thereof, the Research Ethics Review Board(MOH-RERB) of the Ministry of Health has determined that according to the National Guidelines September 2019, for research involving humans in the Republic of South Sudan, the activity highlighted therein, meets the requirement and criteria for approval for the implementation of the research activity and exempted from its MOH-RERB oversight.

The MOH-RERB requires you to comply with all institutional guidelines, rules, and regulations and with the tenets of the code. MOH-RERB reserves the right to conduct compliance visit to your research site without previous notification.

MINISTRY OF HEALTH, RESEARCH ETHICS REVIEW BOARD
MOH-RERB
06 FEB 2023
JUBA SOUTH SUDAN

Amanya Jacob, MPH-SMU, PhD Cand. NU,

D/Director Research, MOH-Juba

Deputy Chairperson, Research Ethics and Review Board, Ministry of Health-Juba, Republic of South Sudan (RERB-MOH)

Cc: U/S MOH-Juba,

Cc: D/G RH Services, MOH-Juba

Cc: D/G PPB & R, MOH-Juba

Cc: D/G SMOH WBGS, NBGS/WS
