# Supplementary material for: Medication nonadherence and associated factors in patients with tuberculosis in Wau, South Sudan: a cross- sectional study using the world health organization multidimensional adherence model
Source: Arch Public Health. 2024 Jul 15;82:107. doi: 10.1186/s13690-024-01339-9 (PMC11250949; doi:10.1186/s13690-024-01339-9)
Supplement: Supplementary file 3 — Supplementary Material 3 [file 13690_2024_1339_MOESM3_ESM.pdf]

INFORMED CONSENT FORM

(For adults research participants aged 18 years and above)

TITLE OF THE STUDY: Elucidation of non-adherence to medication through a multi-dimensional approach: a case of tuberculosis treatment in Western Bahr El Ghazal State, Wau -South Sudan

INTRODUCTION: My name is **Peter Michael Marin** a researcher from **Makerere University** (Uganda) **and University of Bahr el Ghazal, college of Public and Environmental Health** (South Sudan), I would like to learn about factors that help patients with TB to strictly follow or not follow their prescribed medication timely as requested by their health workers among patients with TB in Wau, WBGS-South Sudan.

PURPOSE OF THE STUDY: This study will investigate the factors and reasons the help the patients with TB to strictly follow or not follow their prescribed medication timely as requested by their health workers. We would like to understand the extent of medication adherence and factors contributing to it. Therefore, your participation is important and **voluntary** to help us to learn more about factors and reasons contributing to medication adherence. The samples and information obtained will be used solely for educational and research purposes.

STUDY PROCEDURES: If you agree to participate in this study, we will ask you few questions (questionnaire) and to provide urine sample during your present at health facility. The collected sample will be analysed and the results will be confiedential and you only be informed (will not be share to your health care provider). After analysis within 3 hours the sample will be discrated in accordance with medical waste disposal guidelines using biohazard disposable bags and finally at incenerator. No further analysis will be conducted.

STUDY DURATION: This study data collection will take 6 months, but you will participate once.

STUDY PARTICIPANTS AND STUDY LOCATION

This study is targeting patients receiving first line TB medication at selected health facilities, who are 18 years and above, mentally sound and able to understand the study requirements and consent to participate. The sample size for this study is 216 patients, the participants will be

interviewed once without prior notice at selected health facility TB units when he/she come for monthly visit and medicine collection.

RISKS/DISCOMFORTS:

There is no known risks associated with this study. However, if you may feel not comfortable for any reasons you can feel free not to answer/or to withdraw from the participation.

BENEFITS OF THE RESEARCH STUDY:

The findings of this research will help healthcare workers and National TB program to improve TB treatment adherence and prevention to you, State, country and the world.

COST:

You will not pay any money for your participation in this study.

COMPENSATION FOR PARTICIPATION IN THE STUDY:

The study will provide you with one bar of washing soap as compensation for your time participating in the study.

QUESTIONS ABOUT THE STUDY:

In case you have any other questions related to the study or to your participation you may contact Mr Peter Michael Marin, the Principal Investigator of the study or may reach him at University of Bahr el Ghazal, College of Public and Environmental Health, Wau or contact +211915848061.

QUESTIONS ABOUT PARTICIPANTS RIGHTS:

Should you have questions related to your rights as a research participant that have not been answered by the Principal Investigator or if you wish to report any concerns about the study you can contact Director General, Ministry of Health, Western Bahr El Ghazal State.

STATEMENT OF INFORMED CONSENT:

You participation in this study is voluntary and you may join on your free will and you have a right to withdraw at any time without penalty. Do you agree to take part in this study?

RESPONDENT ANSWER?

Yes ………………………..

No ………………………...

STATEMENT OF CONSENT

I understand the procedures described above. My questions have been answered to my satisfaction and I agree voluntary to take part in this study and I reserve the right to withdraw at any time if I will.

Name of research participant……………………………………………Age………………..

Signature/thumbprint

Date ………………………
