# Supplementary material for: Medication nonadherence and associated factors in patients with tuberculosis in Wau, South Sudan: a cross- sectional study using the world health organization multidimensional adherence model
Source: Arch Public Health. 2024 Jul 15;82:107. doi: 10.1186/s13690-024-01339-9 (PMC11250949; doi:10.1186/s13690-024-01339-9)
Supplement: Supplementary file 4 — Supplementary Material 4 [file 13690_2024_1339_MOESM4_ESM.pdf]

Questionnaire about "TB Treatment non-adherence and its associated factors" in WBG State - South Sudan.

Participant code #:	Location (**GPS**):	Patient registration Book#:
**Section(1): Socio-economics related factors information:**<br>SE/A. Please place (×) or tick (√) a number which best represents your answer.		
S/No	Questions	
SE/A1	Age	1. 18-28 years ☐<br>2. 28-38 years ☐<br>3. 38-48 years ☐<br>4. above 48 years ☐
SE/A2	Sex	1. Male ☐<br>2. Female ☐
SE/A3	Marital status	1. Single ☐<br>2. Married ☐<br>3. Widowed ☐<br>4. Divorced ☐
SE/A4	Education level	1. None ☐<br>2. Primary/basic ☐<br>3. Secondary ☐<br>4. Post-secondary/University ☐
SE/A5	Occupation	…………………………………………………………………
SE/A6	Ethnicity	…………………………………………………………………
SE/A7	Distance to health facility	1. 0-5 km ☐<br>2. More than 5 km ☐
SE/A8	Residency	1. Urban area ☐<br>2. Rural area ☐
SE/A9	Household assets based ownership	1. Motor vehicle ☐ Yes ☐ No
SE/A10		2. Motorcycle ☐ Yes ☐ No
SE/A11		3. Bicycle ☐ Yes ☐ No
SE/A12		4. Canoe/boat ☐ Yes ☐ No
SE/A13		5. TV satellite ☐ Yes ☐ No
SE/A14		6. Radio ☐ Yes ☐ No
SE/A15		7. Phone ☐ Yes ☐ No
SE/A16		8. Computer/Lab top ☐ Yes ☐ No
SE/A17		9. Refrigerator ☐ Yes ☐ No
SE/A18		10. Fan ☐ Yes ☐ No
SE/A19		11. Air conditioner ☐ Yes ☐ No
SE/A20	Do you pay for your transport when coming for follow-up	1. Yes ☐<br>2. No ☐
*	Family support (Don't answer now)	1. Present ☐<br>2. Absent ☐
*	Social support (Don't answer now)	1. Present ☐<br>2. Absent ☐

TB type, Treatment history and phase, treatment supporter			
S/No	Questions		
SE/A21	Type of TB	1. Pulmonary TB<br>2. Extra-pulmonary TB<br>3. TB-HIV	☐<br>☐<br>☐
SE/A22	TB treatment history	1. New<br>2. Retreatment after relapse<br>3. Retreatment after failure<br>4. Retreatment after lost to follow up	☐<br>☐<br>☐<br>☐
SE/A23	Relationship of Treatment supporter	1. Family member<br>2. Non-family member<br>3. Missing	☐<br>☐<br>☐
SE/A24	HIV and antiretroviral ART therapy status	1. HIV positive<br>2. HIV negative<br>3. HIV positive, not on ART	☐<br>☐<br>☐

**Section(2): Therapy related dimension factors :**<br>**Th/G**. Please place (×) or tick (√) a number which best represents your answer.			
S/No	Questions		
Th/G1	Type of regimen	1. (2)HRZE/(4)RH (1st line regimen)<br>2. (2)SHRZE/(1)HRZE/(5)RHE<br>3. Others	☐<br>☐<br>☐
Th/G2	*Previous treatment failure	1. Yes<br>2. No	☐<br>☐
Th/G3	Have you informed how to managed side effect?	1. Yes<br>2. No	☐<br>☐
Th/G4	Current treatment phase	1. Intensive<br>2. Continuation<br>3. Month #	☐<br>☐<br>☐

**Section(3): Condition related dimension factors:**<br>**Co/H**. Please place (×) or tick (√) a number which best represents your answer.			
S/No	Questions		
Co/H1	Severity of symptoms	1. Yes<br>2. No	☐<br>☐
Co/H2	*Psychological distress (Don't answer now)	1. Present<br>2. Absent	☐<br>☐
Co/H3	*Alcohol disorder (Don't answer now)	1. Present<br>2. Absent	☐<br>☐
Co/H4	*Smoking history	1. Yes<br>2. No	☐<br>☐

Section(4): **Healthcare team and system related dimension factors:**<br>**HCT/J**. Please place (×) or tick (√) a number which best represents your answer.			
S/No	Questions		
HCT/J1	Type of DOT service	1. Facility based-DOT<br>2. Home based-DOT	☐<br>☐
HCT/J2	Waiting time to receive service	1. Less than 60 minutes<br>2. More than 60 minutes	☐<br>☐
HCT/J3	Last counselling and health education	1. Last month<br>2. More than month	☐<br>☐
HCT/J4	*Relationship with healthcare workers<br>(Don't answer now)	1. Satisfied<br>2. Dissatisfied	☐<br>☐
HCT/J5	Are you receiving your TB drugs every month?<br>(Anti-TB drugs supply available)	1. Yes<br>2. No	☐<br>☐

**Section (5): Patient related dimension factors (Knowledge about Tuberculosis)**<br>**P/B**. Please place (×) or tick (√) a number which best represents your answer.			
S/No	Questions		
P/B1	How is TB transmitted from one person to another?	1. Through cough or sneeze of sick person<br>2. Through handshakes<br>3. Don't know	☐<br>☐<br>☐
P/B2	Would a person with TB eat together with other family members?	1. Yes<br>2. No	☐<br>☐
P/B3	Do family contacts "members" need to be test for TB?	1. Yes<br>2. No	☐<br>☐
P/B4	There is effective medical treatment for TB?	1. Yes<br>2. No	☐<br>☐
P/B5	TB disease is curable, If treated without missing taking all prescribed drug.	1. Yes<br>2. No	☐<br>☐
P/B6	What is the duration of treatment required to cure TB?	1. One to two(1-2) months<br>2. Six to eight (6-8) months<br>3. Rest of your life<br>4. Don't know	☐<br>☐<br>☐<br>☐
P/B7	Can TB treatment be stopped once you feel better even without completing taking all drugs?	1. Yes<br>2. No	☐<br>☐
P/B8	Traditional drugs are effective to cure TB?	1. Yes<br>2. No	☐<br>☐
P/B9	Witchcraft, devil spirits and curse can cause TB?	1. Yes<br>2. No	☐<br>☐
P/B10	The main signs and symptoms of TB are cough more than 2 weeks, coughing up blood and weight loss.	1. Yes<br>2. No	☐<br>☐
P/B11	TB can be prevented through covering mouth and nose when coughing or sneezing	1. Yes<br>2. No	☐<br>☐

**Section (5)**: **Patient related dimension factors** (**Patient perspective towards TB related stigma)**<br>**P/C.** Please ask the participants place (×) or tick (√) a number which best represents your answer.					
S/No	Questions (patient Self-reported)	Strongly disagree (0)	Disagree (1)	Agree (2)	Strongly Agree (3)
P/C1	Some people who have TB feel hurt of how others react to knowing they have TB.				
P/C2	Some people who have TB lose friends when they share with them they have TB.				
P/C3	Some people who have TB feel alone.				
P/C4	Some people who have TB keep their distance from others to avoid spreading TB germs.				
P/C5	Some people who have TB are afraid to tell those outside their family that they have TB.				
P/C6	Some people who have TB are afraid of going to TB clinics because others may see them there.				
P/C7	Some people who have TB are afraid to tell others that they have TB because others may think that they also have AIDS.				
P/C8	Some people who have TB feel guilty because their family has the burden of caring for them.				
P/C9	Some people who have TB will choose carefully who they tell about having TB.				
P/C10	Some people who have TB feel guilty for getting TB because of their smoking, drinking, or other careless behaviors.				
P/C11	Some people who have TB are worried about having AIDS.				
P/C12	Some people who have TB are afraid to tell their family that they have TB.				

**Section (5)**: **Patient related dimension factors: Psychological distress Question (Kessler Psychological Distress Scale, K-10):**<br>**P/D**. Please ask the participants place (×) or tick (√) a number which best represents your answer.						
S/No	Questions	All the time	Most of the time	Some of the time	A Little of the time	None of the time
P/D1	In the past 4 weeks, about how often did you feel tired out for no good reason?					
P/D2	In the past 4 weeks, about how often did you feel nervous?					

P/D3	In the past 4 weeks, about how often did you feel so nervous that nothing could calm you down?					
P/D4	In the past 4 weeks, about how often did you feel hopeless?					
P/D5	In the past 4 weeks, about how often did you feel restless or fidgety?					
P/D6	In the past 4 weeks, about how often did you feel so restless you could not sit still?					
P/D7	In the past 4 weeks, about how often did you feel depressed?					
P/D8	In the past 4 weeks, about how often did you feel that everything was an effort?					
P/D9	In the past 4 weeks, about how often did you feel so sad that nothing could cheer you up?					
P/D10	In the past 4 weeks, about how often did you feel worthless?					

**Section (5)**: **Patient related dimension factors: The Tobacco Use History**<br>**P/D**. Please place (×) or tick (√) a number which best represents your answer.		
S/No	Questions	
P/D11	Do you currently use one or more of the following tobacco products (cigarettes, snuff, chewing tobacco, cigars, etc.)?	1. Yes ☐<br>2. No ☐
P/D12	In the last month, how often have you used one or more of the following tobacco products (cigarettes, snuff, chewing tobacco, cigars, etc.)?	1. once ☐<br>2. twice ☐<br>3. weekly ☐<br>4. almost daily ☐<br>5 daily ☐

**Section (5)**: **Patient related dimension factors:**<br>**P/D. The Alcohol Use Disorders Identification Test: Interview Version**<br>Read questions as written. Record answers carefully. Begin the AUDIT by saying "Now I am going to ask you some questions about your use of alcoholic beverages during this past year." Explain what is meant by "alcoholic beverages" by using local examples of local beer, wine, etc. Code answers in terms of "standard drinks". Place the correct answer number in the box at the right.		
	**P/D1.** How often do you have a drink containing alcohol?<br>0 = Never [Skip to Qs 9-10]<br>1= Monthly or less<br>2 = 2 to 4 times a month<br>3 = 2 to 3 times a week<br>4 = 4 or more times a week<br>☐	**P/D2.** How many drinks containing alcohol do you have on a typical day when you are drinking?<br>0 = 1 or 2<br>1 = 3 or 4<br>2 = 5 or 6<br>3 = 7, 8, or 9<br>4 = 10 or more<br>☐

	**P/D3.** How often do you have six or more drinks on one occasion?<br>0 = Never<br>1 = Less than monthly<br>2 = Monthly<br>3 = Weekly<br>4 = Daily or almost daily<br>Skip to Questions 9 and 10 if Total Score for Questions 2 and 3 = 0	**P/D4.** How often during the last year have you found that you were not able to stop drinking once you had started?<br>0 = Never<br>1 = Less than monthly<br>2 = Monthly<br>3 = Weekly<br>4 = Daily or almost daily
	**P/D5.** How often during the last year have you failed to do what was normally expected from you because of drinking?<br>0 = Never<br>1= Less than monthly<br>2 = Monthly<br>3 = Weekly<br>4 = Daily or almost daily	**P/D6.** How often during the last year have you needed a first drink in the morning to get yourself going after a heavy drinking session?<br>0 = Never<br>1 = Less than monthly<br>2 = Monthly<br>3 = Weekly<br>4 = Daily or almost daily
	**P/D7.** How often during the last year have you had a feeling of guilt or remorse after drinking?<br>0 = Never<br>1 = Less than monthly<br>2 = Monthly<br>3 = Weekly<br>4 = Daily or almost daily	**P/D8.** How often during the last year have you been unable to remember what happened the night before because you had been drinking?<br>0 = Never<br>1 = Less than monthly<br>2 = Monthly<br>3 = Weekly<br>4 = Daily or almost daily
	**P/D9.** Have you or someone else been injured as a result of your drinking?<br>0 = No<br>2 = Yes, but not in the last year<br>4 = Yes, during the last year	**P/D10.** Has a relative or friend or a doctor or another health worker been concerned about your drinking or suggested you cut down?<br>0 = No<br>2 = Yes, but not in the last year<br>4= Yes, during the last year
Sum up specific items values to calculate total, then record here		
NB: If total is greater than recommended cut-off, consult User's Manual (*Recommended cut-off scores for person less than 65 is 8 and for person above 65 is 7*)		

**Section(4): Healthcare team and system related dimension factors:**<br>**HCP- Patient Communication:** These people including doctors, nurses, TB community workers, lab technician & TB volunteers.<br>**HCT/E**. Please place (×) or tick (√) a number which best represents your answer					
S/No	Questions/Response	Strongly disagree (0)	Disagree (1)	Agree (2)	Strongly Agree (3)
HCT/E1	Health worker told me what the possible side-effects of each of the TB drug are?				
HCT/E2	Health worker told me what treatment would do				
HCT/E3	Health worker told me how the TB treatment would be				
HCT/E4	Health worker told me the changes to expect in my health when taking TB medication				
HCT/E5	Treatment procedure was clearly explained by the Health worker				
S/No	Question<br>Circle a number which represent the appropriate response	Very dissatisfied (0)	Somewhat dissatisfied (1)	Somewhat satisfied (2)	Very satisfied (3)
HCT/E6	In general, how satisfied are you with the overall support you get from the HCP?				

**Section(I): Socio-economics related factors information: Social and family support**<br>**SE/F**. Please place (×) or tick (√) a number which best represents your answer					
S/No	Questions	Not at all (0)	A little (1)	Somewhat (2)	A lot (3)
Do you tell some of the following people that you are having TB treatment?					
SE/F1	Family				
SE/F2	Friends				
SE/F3	Neighbor(s)				
SE/F4	Treatment supporter/peer group				
SE/F5	NGOs/Government				
To what extent do the following people help you to remember to take TB medication?		Not at all (0)	A little (1)	Somewhat (2)	A lot (3)
SE/F6	Family				
SE/F7	Friends				
SE/F8	Neighbor(s)				
SE/F9	Treatment supporter/peer group				

SE/F10	NGOs/Government				
To what extent do the following people provide you emotional support (e.g. acceptance, freedom to talk openly about your health, care, ability to confide in, ability to share grief, and the like)?		Not at all (0)	A little (1)	Somewhat (2)	A lot (3)
SE/F11	Family				
SE/F12	Friends				
SE/F13	Neighbor(s)				
SE/F14	Treatment supporter/peer group				
SE/F15	NGOs/Government				
To what extent do the following people provide you tangible support (e.g. finances, transportation, house chore, housing, clothing, food supplies, medical supplies, children education and the like)?		Not at all (0)	A little (1)	Somewhat (2)	A lot (3)
SE/F16	Family				
SE/F17	Friends				
SE/F18	Neighbor(s)				
SE/F19	Treatment supporter/peer group				
SE/F20	NGOs/Government				
To what extent do the following people provide you information support (e.g. advise, guidance, feedback or information on a variety of issues and the like)?		Not at all (0)	A little (1)	Somewhat (2)	A lot (3)
SE/F21	Family				
SE/F22	Friends				
SE/F23	Neighbor(s)				
SE/F24	Treatment supporter/peer group				
SE/F25	NGOs/Government				
In general, how satisfied are you with the overall support you get from the following?		Not at all (0)	A little (1)	Somewhat (2)	A lot (3)
SE/F26	Family				
SE/F27	Friends				
SE/F28	Neighbor(s)				
SE/F29	Treatment supporter/peer group				
SE/F30	NGOs/Government				

Thank you for your time!
