# Supplementary material for: Medication nonadherence and associated factors in patients with tuberculosis in Wau, South Sudan: a cross- sectional study using the world health organization multidimensional adherence model
Source: Arch Public Health. 2024 Jul 15;82:107. doi: 10.1186/s13690-024-01339-9 (PMC11250949; doi:10.1186/s13690-024-01339-9)
Supplement: Supplementary file 5 — Supplementary Material 5 [file 13690_2024_1339_MOESM5_ESM.pdf]

TB treatment Adherence Measurement Tools Questionnaire

Patient code # ……………………………… **Location (Health facility)** ………………………………………………
Date of interview …………………………………. **Time** …………………………………………………… a.m/p.m

**1. IsoScreen Test (Urine isoniazid metabolite testing)**	
**Questions**	
Urine sample taken (keep the sample carefully and follow hazardous prevention procedures. Keep sample at cool box).	1. Yes ☐<br>2. No ☐
What is the colour of urine reaction after performing the test?	1. Purple/blue (Positive) ☐<br>2. Green (Equivocal) ☐<br>3. Yellow (Negative) ☐
**Interpretation of IsoScreen result:**	a. Adherence ☐<br>b. Suboptimal adherence ☐<br>c. Nonadherence ☐

2. Visual Analogue Scale (VAS)

How many of your TB tablet did you **missed** in the last **30** days? ☐ ☐ **%**

(not single dose missed) **(not single dose taken)**

0% ⟷ **50%** ⟷ **100%**

Interpretation of the VAS result: (equal/or more than 10% is considered nonadherence)

1. Adherence ☐ 2. Nonadherence ☐

3. What are the possible reasons of the missing doses?

----

4. Final Treatment outcomes (after completion of 6 months of treatment)

What is the final treatment outcome?<br>(As documented in the **patient treatment card**)	1. Cured ☐<br>2. Treatment completed ☐<br>3. Treatment failed ☐<br>4. Lost to follow up ☐<br>5. Died ☐

Final Remarks
